# Digital Holographic Multimodal Cross-Sectional Fluorescence and Quantitative Phase Imaging System

**DOI:** 10.1038/s41598-020-64028-x

**Published:** 2020-05-15

**Authors:** Manoj Kumar, Xiangyu Quan, Yasuhiro Awatsuji, Yosuke Tamada, Osamu Matoba

**Affiliations:** 1 Graduate School of System Informatics, Kobe University, Rokkodai 1-1, Nada, Kobe, 657-8501 Japan; 2 Faculty of Electrical Engineering and Electronics, Kyoto Institute of Technology, Matsugasaki, Sakyo-ku, Kyoto, 606-8585 Japan; 3 School of Engineering, Utsunomiya University, 7-1-2 Yoto, Utsunomiya, 321-8585 Japan; 4 National Institute for Basic Biology, 38 Nishigonaka, Myodaiji, Okazaki, 444-8585 Japan; 5 School of Life Science, SOKENDAI (The Graduate University for Advanced Studies), 38 Nishigonaka, Myodaiji, Okazaki, 444-8585 Japan; 6 Center for Optical Research and Education (CORE), Utsunomiya University, 7-1-2 Yoto, Utsunomiya, 321-8585 Japan

**Keywords:** Imaging and sensing, Biophotonics

## Abstract

We present a multimodal imaging system based on simple off-axis digital holography, for simultaneous recording and retrieval of cross-sectional fluorescence and quantitative phase imaging of the biological specimen. Synergism in the imaging capabilities can be achieved by incorporating two off-axis digital holographic microscopes integrated to record different information at the same time. The cross-sectional fluorescence imaging is realized by a common-path configuration of the single-shot off-axis incoherent digital holographic system. The quantitative phase imaging, on the other hand, is achieved by another off-axis coherent digital holographic microscopy operating in transmission mode. The fundamental characteristics of the proposed multimodal system are confirmed by performing various experiments on fluorescent beads and fluorescent protein-labeled living cells of the moss *Physcomitrella patens* lying at different axial depth positions. Furthermore, the cross-sectional live fluorescence and phase imaging of the fluorescent beads are demonstrated by the proposed multimodal system. The experimental results presented here corroborate the feasibility of the proposed system and indicate its potential in the applications to analyze the functional and structural behavior of biological cells and tissues.

## Introduction

Over the past decade, multimodal systems based on quantitative phase imaging in combination with fluorescence imaging have been developed considerably due to its several advantages^1–12^. The phase imaging reveals the structural information by exploiting the optical path-length shifts through the biological specimen, while fluorescence imaging provides functional details of the specific molecules of interest in the biological specimen. Therefore, a hybrid multimodal imaging system comprising both these systems offers to precisely visualize and delineate structural and functional information in the biological specimen on a single platform at the same time.

The quantitative phase imaging technique^13–16^ is an emerging powerful technology of a new paradigm in general imaging and biomedical applications that provides quantitative information including the structure and dynamics of the transparent specimens, which is not easily obtained with the conventional optical imaging techniques. The technique employs the principle of optical interferometry to measure the optical field (i.e. amplitude and phase information). In recent years, quantitative phase imaging has received substantial interest and opened the door for quantifying dynamic structural information of biological cells with nanoscale sensitivity. Popescu *et al*.^17^ reported the use of phase imaging for monitoring cell growth, characterizing cellular motility, and investigating the subcellular motions of living cells. The technique has also found applications for various investigations of cells and tissues including measurement of 3D profiling and tracking^15^, refractive index^18^, spectral dispersion^19^, optical path length^20^, dry mass localization^21^, and optimum focus determinations^22^, etc.

In order to study the functional information of a biological sample including cellular and microbiological investigations, fluorescence microscopy is a crucial technique. This technique has been investigated for the study of microbiological processes^23,24^, cellular and molecular identification^25–27^, and rapid detection and diagnosis^28^. Since fluorescence is the most important source of enhancing contrast in biological imaging and the fluorescence images can be acquired from a sample labeled with fluorophores that encode the molecular specificity of the specimen. Most fluorescence imaging techniques involve sectioning, such as laser scanning confocal microscopy^29,30^ or spinning disk confocal microscopy^31^, which are time-consuming processes to obtain images of the 3D field. Further, efforts of adopting digital holography to fluorescence microscopy have been realized. However, these efforts make the system more complicated and the whole process becomes time-consuming^32,33^. Recently, holographic fluorescence microscopy has been possible due to the advent of Fresnel incoherent correlation holography (FINCH)^34–37^, as fluorescence light is naturally incoherent^38^. FINCH is based on the self-interference principle stated that two or more beams originating from the same source point are mutually coherent and interfere to form the in-line digital hologram^37^. In FINCH, the incident wave originated from each object point is allowed to split into two waves with different curvatures by using an appropriate optical component (say an appropriate pattern, e.g., a grating pattern, displayed on a spatial light modulator) and an interference pattern is produced by the recombination of these waves at an appropriate overlapped region in the image sensor. The continuous progress in the field of FINCH promises new applications. However, FINCH is an inline-holography and thus the phase-shifting method is required to reduce unwanted terms such as DC and conjugated term. In this line of research, we have proposed a single-shot common-path off-axis FINCH system^39,40^ for imaging of biological samples. This configuration of modified FINCH called common-path off-axis incoherent digital holography is accomplished by embedding a focusing lens with a diffraction grating onto a phase-mode spatial light modulator (SLM) to generate two slightly different wavefronts and different propagation directions to form a digital hologram at the image sensor.

A combination of holographic fluorescence microscopy with quantitative phase imaging to obtain the fluorescence and the phase information will open up new possibilities of an exciting frontier to extract more information about the targeted biological specimen. Recently, we have proposed a multimodal imaging system by incorporating the common-path configurations of both the digital holographic microscopes, for simultaneous recording and retrieval of 3D fluorescence and phase imaging of a biological specimen^40^. This system is very stable due to the common-path geometry for both the fluorescence and phase imaging. In the phase imaging, however, it is not easy to control the fringe frequency of the off-axis hologram to create a tilted plane reference wave via the transmittance through a pinhole. The intensity ratio between the object and the reference waves could not be controlled. This degrades the ability of quantitative phase imaging. In this paper, a multimodal digital holographic system is developed by comprising the common-path incoherent digital holographic microscope, for retrieving the 3D fluorescence imaging, with the two-arm off-axis coherent digital holographic microscope, for retrieving the phase imaging. In this configuration, the two-arm off-axis coherent digital holographic microscope implementation is easily installed in the multimodal digital holographic system. This is the first paper, to our best knowledge, that evaluates the quantitative phase accuracy in the multimodal digital holographic microscopes as well as dynamic phase measurement of floating fluorescence beads in the 3D field. The multimodal system could, indeed, have the capability to provide high-contrast functional imaging along with structural details of the biological specimen on a common platform from which it is possible to extract important intrinsic biophysical parameters. The present system has the ability not only to provide the 2D (*x*, *y*) information but also depth information (*z*-direction) of the fluorescent objects in a single-shot without scanning. It should be noted that the incoherent digital holographic system provides good imaging results for sparse objects and the technique is capable of retrieval cross-sectional information for the objects separated by an axial depth of about 250 micrometers.

## Methodology

**Figure 1:**
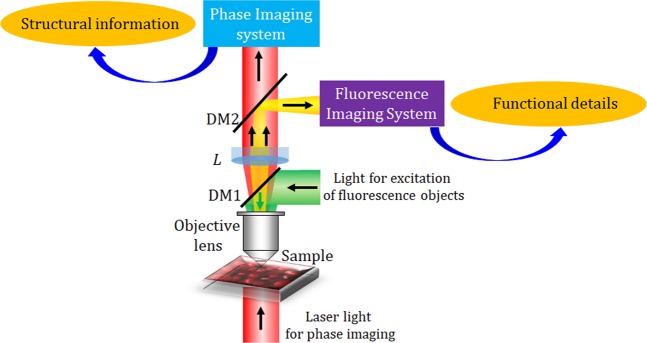
Schematic of the proposed multimodal system for recording off-axis fluorescence and phase digital holography.

In this work, we have presented a common-path off-axis incoherent digital holographic microscope integrated with another off-axis coherent digital holographic microscope for simultaneous measurement of cross-sectional fluorescence imaging and quantitative phase of fluorescent objects. The schematic presentation of the proposed multimodal system is depicted in Fig. 1, where the multimodal system allowing to retrieve simultaneously the structural information by the phase imaging system and the functional details by the fluorescence imaging system of the specimen under study. In the following subsections, the descriptions of both the systems are presented.

### Common-path off-axis incoherent digital holography for cross-sectional fluorescence imaging

**Figure 2:**
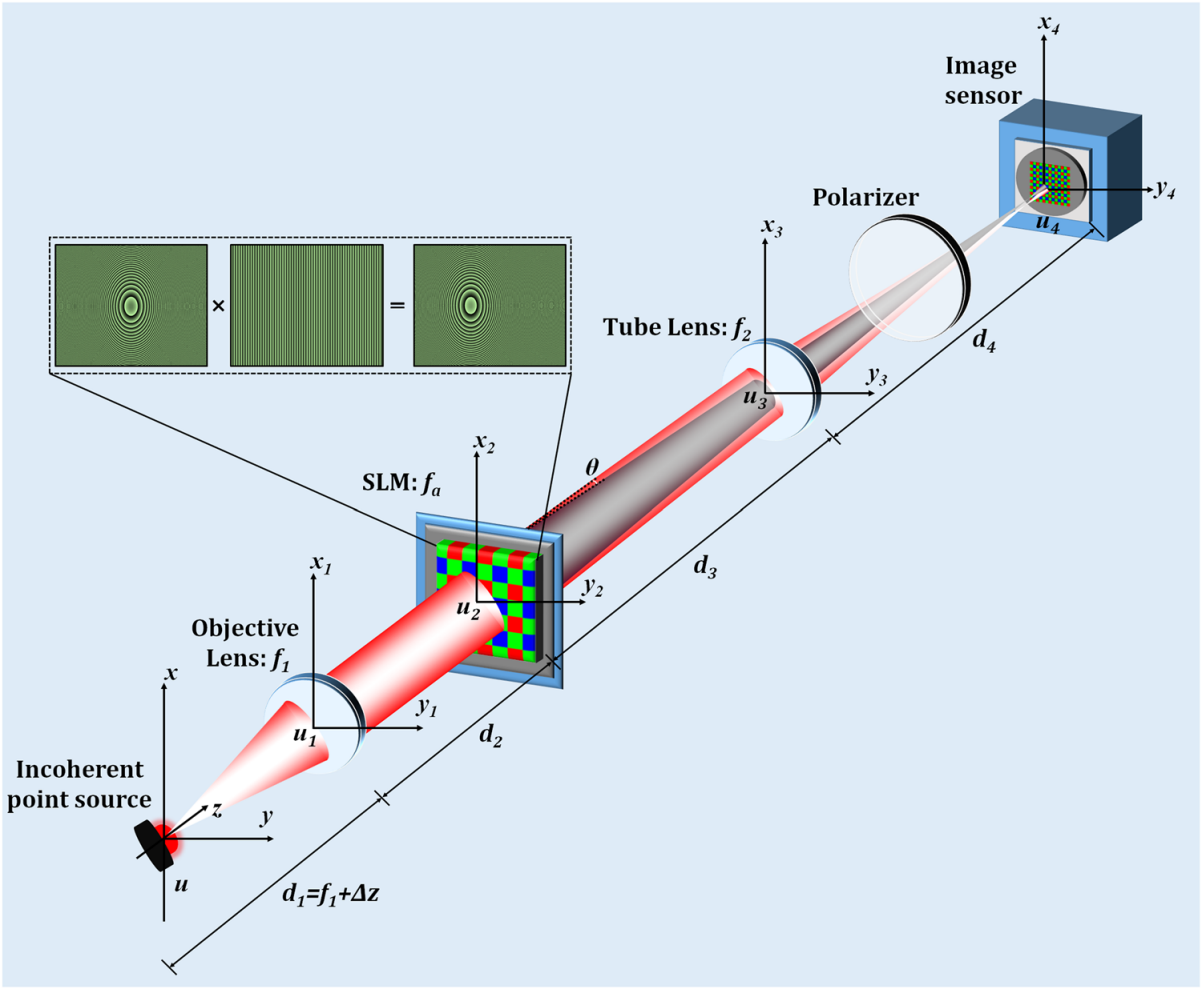
Schematic of the common-path off-axis incoherent digital holographic setup. Top panels indicate the phase pattern displayed on the SLM.

Figure 2 shows the schematic of the single-shot common-path off-axis incoherent digital holographic microscope. The system is accomplished by embedding a focusing lens with a diffraction grating onto a phase-mode spatial light modulator (SLM) which splits the incident fluorescent light from the fluorescent object into two light waves with slightly different propagation directions in order to achieve off-axis interference. These two wavefronts interfered at the image sensor plane and form a fluorescent digital hologram.

The mathematical description of the proposed setup, by considering the one-dimensional case, is as follows. Here, we assume that the point source is located at (*x*_0_, 0, −Δ*z*). The optical wavefield by a point source *δ*(*x*-*x*_0_) obtained by the Fresnel propagation from *x* plane to *x*_1_ plane with a distance of *d*_1_ (=*f*_1_ + Δ*z*) is described as

1 $${u}_{1}({x}_{1})={\int }_{-\infty }^{\infty }\delta (x-{x}_{0})\exp \left[i\frac{\pi }{\lambda {d}_{1}}{({x}_{1}-x)}^{2}\right]dx=\exp \left[i\frac{\pi }{\lambda {d}_{1}}{({x}_{1}-{x}_{0})}^{2}\right]$$

where, *δ* is the Dirac delta function. Then, a lens function with a focal length of *f*_1_ is multiplied

2 $${u}_{11}({x}_{1})=\exp \left[i\frac{\pi }{\lambda {d}_{1}}{({x}_{1}-{x}_{0})}^{2}\right]\exp \left(-i\frac{\pi }{\lambda {f}_{1}}{x}_{1}^{2}\right)=a\exp \left(-i\frac{2\pi }{\lambda {d}_{1}}{x}_{0}{x}_{1}\right)\exp \left(i\frac{\pi }{\lambda {d}_{11}}{x}_{1}^{2}\right)$$

where

3 $$\frac{1}{{d}_{11}}=\frac{1}{{d}_{1}}-\frac{1}{{f}_{1}};\,a=\exp \left(i\frac{\pi }{\lambda {d}_{1}}{x}_{0}^{2}\right)$$

Another Fresnel propagation from *x*_1_ plane to *x*_2_ plane is described as

4 $$\begin{array}{lll}{u}_{2}({x}_{2}) & = & a{\int }_{-\infty }^{\infty }\exp \left(-i\frac{2\pi }{\lambda {d}_{1}}{x}_{0}{x}_{1}\right)\exp \left(i\frac{\pi }{\lambda {d}_{11}}{x}_{1}^{2}\right)\exp \left[i\frac{\pi }{\lambda {d}_{2}}{({x}_{2}-{x}_{1})}^{2}\right]d{x}_{1}\\  & = & {a}_{1}\exp \left(-i\frac{2\pi }{\lambda }\frac{{d}_{21}}{{d}_{1}{d}_{2}}{x}_{0}{x}_{2}\right)\exp \left(i\frac{\pi }{\lambda {d}_{22}}{x}_{2}^{2}\right)\,\end{array}$$

where

5 $$\begin{array}{l}\frac{1}{{d}_{21}}=\frac{1}{{d}_{11}}+\frac{1}{{d}_{2}};\,\frac{1}{{d}_{22}}=\frac{1}{{d}_{2}}-\frac{{d}_{21}}{{d}_{2}^{2}};{\rm{or}}\,{d}_{22}={d}_{2}+{d}_{11};\\ {a}_{1}=\exp \left(i\frac{\pi }{\lambda {d}_{1}}{x}_{0}^{2}\right)\exp \left(-i\frac{\pi {d}_{21}}{\lambda {d}_{1}^{2}}{x}_{0}^{2}\right)=\exp \left[i\frac{\pi }{\lambda }\left(\frac{1}{{d}_{1}}-\frac{{d}_{21}}{{d}_{1}^{2}}\right){x}_{0}^{2}\right]\end{array}$$

At SLM (*x*_2_ plane), the object light is multiplied by a diffraction grating and a lens function with a focal length of *f*_*a*_ in a phase-mode SLM.

6 $$\begin{array}{lll}{u}_{21}({x}_{2}) & = & {a}_{1}\exp \left(-i\frac{2\pi }{\lambda }\frac{{d}_{21}}{{d}_{1}{d}_{2}}{x}_{0}{x}_{2}\right)\exp \left(i\frac{\pi }{\lambda {d}_{22}}{x}_{2}^{2}\right)\exp \left(-i\frac{\pi }{\lambda {f}_{a}}{x}_{2}^{2}\right)\exp \left(i\frac{2\pi }{\lambda }\,\sin \,\theta {x}_{2}\right)\\  & = & {a}_{1}\exp \left[-i\frac{2\pi }{\lambda }\left(\frac{{x}_{0}}{{d}_{t}}-\,\sin \,\theta \right){x}_{2}\right]\exp \left(i\frac{\pi }{\lambda {d}_{23}}{x}_{2}^{2}\right)\,\end{array}$$

where

7 $$\frac{1}{{d}_{t}}=\frac{{d}_{21}}{{d}_{1}{d}_{2}};\,{\rm{or}}\,{d}_{t}=\frac{{{\rm{d}}}_{1}({d}_{11}+{d}_{2})}{{d}_{11}};\frac{1}{{d}_{23}}=\frac{1}{{d}_{22}}-\frac{1}{{f}_{a}}$$

Another Fresnel propagation from *x*_2_ plane to *x*_3_ plane is implemented.

8 $$\begin{array}{lll}{u}_{3}({x}_{3}) & = & {a}_{1}{\int }_{-\infty }^{\infty }\exp \left[-i\frac{2\pi }{\lambda }\left(\frac{{x}_{0}}{{d}_{t}}-\,\sin \,\theta \right){x}_{2}\right]\exp \left(i\frac{\pi }{\lambda {d}_{23}}{x}_{2}^{2}\right)\exp \left[i\frac{\pi }{\lambda {d}_{3}}{({x}_{3}-{x}_{2})}^{2}\right]d{x}_{2}\\  & = & {}_{2}\exp \left(i\frac{\pi }{\lambda {d}_{32}}{x}_{3}^{2}\right)\exp \left[-i\frac{2\pi }{\lambda {d}_{t1}}({x}_{0}-{d}_{t}\,\sin \,\theta ){x}_{3}\right]\end{array}$$

where

9 $$\begin{array}{l}\frac{1}{{d}_{31}}=\frac{1}{{d}_{23}}+\frac{1}{{d}_{3}};\,\frac{1}{{d}_{32}}=\frac{1}{{d}_{3}}-\frac{{d}_{31}}{{d}_{3}^{2}};\,{\rm{or}}\,{d}_{32}={d}_{3}+{d}_{23};\,\frac{1}{{d}_{t1}}=\frac{{d}_{31}}{{d}_{t}{d}_{3}}\,{\rm{or}}\,{d}_{t1}=\frac{{d}_{1}({d}_{2}+{d}_{11})({d}_{3}+{d}_{23})}{{d}_{23}{d}_{11}};\,{\rm{or}}\,\\ \,{a}_{2}={a}_{1}\exp \left[-i\frac{\pi {d}_{31}}{\lambda }{\left(\frac{{x}_{0}}{{d}_{t}}-\sin \theta \right)}^{2}\right]=\exp \left\{i\frac{\pi }{\lambda }\left[\left(\frac{1}{{d}_{1}}-\frac{{d}_{21}}{{d}_{11}^{2}}\right){x}_{0}^{2}-{d}_{31}{\left(\frac{{x}_{0}}{{d}_{t}}-\sin \theta \right)}^{2}\right]\right\}\end{array}$$

The object wave is multiplying by a lens function with a focal length of *f*_2_

10 $$\begin{array}{lll}{u}_{31}({x}_{3}) & = & {a}_{2}\exp \left(i\frac{\pi }{\lambda {d}_{32}}{x}_{3}^{2}\right)\exp \left[-i\frac{2\pi }{\lambda {d}_{t1}}({x}_{0}-{d}_{t}\,\sin \,\theta ){x}_{3}\right]\exp \left(-i\frac{\pi }{\lambda {f}_{2}}{x}_{3}^{2}\right)\\  & = & {a}_{2}\exp \left(i\frac{\pi }{\lambda {d}_{41}}{x}_{3}^{2}\right)\exp \left[-i\frac{2\pi }{\lambda {d}_{t1}}({x}_{0}-{d}_{t}\,\sin \,\theta ){x}_{3}\right]\end{array}$$

where

11 $$\frac{1}{{d}_{41}}=\frac{1}{{d}_{32}}-\frac{1}{{f}_{2}}$$

The Fresnel propagation from *x*_3_ plane to *x*_4_ plane is obtained as

12 $$\begin{array}{lll}u(x;\,\sin \,\theta ,{f}_{a}) & = & {a}_{2}{\int }_{-\infty }^{\infty }\exp \left(i\frac{\pi }{\lambda {d}_{41}}{x}_{3}^{2}\right)\exp \left[-i\frac{2\pi }{\lambda {d}_{t1}}({x}_{0}-{d}_{t}\,\sin \,\theta ){x}_{3}\right]\exp \left[i\frac{\pi }{\lambda {d}_{4}}{({x}_{4}-{x}_{3})}^{2}\right]d{x}_{3}\\  & = & {a}_{3}\exp \left[-i\frac{2\pi }{\lambda }{d}_{52}\left(\frac{{d}_{11}}{{d}_{1}}\frac{{x}_{0}}{{d}_{2}+{d}_{11}}-\,\sin \,\theta \right){x}_{4}\right]\exp \left(i\frac{\pi }{\lambda {d}_{51}}{x}_{4}^{2}\right)\end{array}$$

where

13 $$\begin{array}{l}\frac{1}{{d}_{42}}=\frac{1}{{d}_{41}}+\frac{1}{{d}_{4}};\,\frac{1}{{d}_{51}}=\frac{1}{{d}_{4}}-\frac{{d}_{42}}{{d}_{4}^{2}};{\rm{or}}\,{d}_{51}={d}_{4}+{d}_{41}={d}_{4}+\frac{{f}_{2}({d}_{3}+{d}_{23})}{{f}_{2}-{d}_{3}-{d}_{23}}\\ \,{d}_{52}=\frac{{d}_{42}{d}_{31}}{{d}_{4}{d}_{3}}=\frac{{d}_{23}({d}_{51}-{d}_{4})}{{d}_{51}({d}_{3}+{d}_{23})}=\frac{{f}_{2}{d}_{23}}{({f}_{2}-{d}_{4})({d}_{23}+{d}_{3})+{d}_{4}{f}_{2}};{d}_{23}=\frac{({d}_{2}+{d}_{11}){f}_{a}}{{f}_{a}-{d}_{2}-{d}_{11}}\\ \,{a}_{3}=\exp \left\{i\frac{\pi }{\lambda }\left[\left(\frac{1}{{d}_{1}}-\frac{{d}_{21}}{{d}_{1}^{2}}\right){x}_{0}^{2}-{d}_{31}{\left(\frac{{x}_{0}}{{d}_{t}}-\sin \theta \right)}^{2}-{d}_{42}\frac{1}{{{d}_{t1}}^{2}}{({x}_{0}-{d}_{t}\sin \theta )}^{2}\right]\right\}\end{array}$$

Equation (12) represents the modulated light wave obtained by the phase pattern (lens function and grating) displayed on to the SLM. The unmodulated light wave reaching at the image sensor is obtained from Eq. (12), by setting *f*_*a*_ = $\infty$ and *θ* = 0, as

14 $$u(x;0,\infty )={a}_{3}^{{\prime} }\exp \left[-i\frac{2\pi }{\lambda }{d}_{52}^{{\prime} }\left(\frac{{d}_{11}}{{d}_{1}}\frac{{x}_{0}}{{d}_{2}+{d}_{11}}\right){x}_{4}\right]\exp \left(i\frac{\pi }{\lambda {d}_{51}^{{\prime} }}{x}_{4}^{2}\right)$$

where

15 $$\begin{array}{lll}{a}_{3}^{{\prime} } & = & \exp \left[i\frac{\pi }{\lambda }\left(\frac{1}{{d}_{1}}-\frac{{d}_{21}}{{d}_{1}^{2}}-\frac{{d}_{31}^{{\prime} }}{{d}_{t}^{2}}-\frac{{d}_{42}^{{\prime} }}{{d{\prime} }_{t1}^{2}}\right){x}_{0}^{2}\right]\\ {d}_{42}^{{\prime} } & = & \frac{{d}_{4}{d}_{41}^{{\prime} }}{{d}_{4}+{d}_{41}^{{\prime} }};{d}_{41}^{{\prime} }=\frac{{f}_{2}{d}_{32}^{{\prime} }}{{f}_{2}-{d}_{32}^{{\prime} }};{d}_{32}^{{\prime} }={d}_{3}+{d}_{22};{d}_{31}^{{\prime} }=\frac{{d}_{3}{d}_{22}}{{d}_{22}+{d}_{3}};{d{\prime} }_{t1}=\frac{{d}_{t}{d}_{3}}{{d}_{31}^{{\prime} }};\end{array}$$

The interference of the modulated and unmodulated light waves is carried out at the faceplate of the image sensor with the help of a linear polarizer. The hologram intensity recorded by the image sensor is represented by

16 $$\begin{array}{l}I(x)={|u(x;0,\infty )+u(x;\sin \theta ,{f}_{a})|}^{2}\\ \,=2+2Re\left\{{a}_{3}^{{\prime} }{a}_{3}^{\ast }\exp \left\{-i\frac{2\pi }{\lambda }\left[({d}_{52}-{d}_{52}^{{\prime} })\left(\frac{{d}_{11}}{{d}_{1}}\frac{{x}_{0}}{{d}_{22}}\right)-{d}_{52}\,\sin \,\theta \right]{x}_{4}\right\}\exp \left[i\frac{\pi }{\lambda }\left(\frac{1}{{d}_{51}}-\frac{1}{{d}_{52}^{{\prime} }}\right){x}_{4}^{2}\right]\right\}\end{array}$$

where

17 $${d}_{51}^{\text{'}}={d}_{4}+\frac{{f}_{2}({d}_{3}+{d}_{22})}{{f}_{2}-{d}_{3}-{d}_{22}};{d}_{52}^{\text{'}}=\frac{{f}_{2}{d}_{22}}{({f}_{2}-{d}_{4})({d}_{22}+{d}_{3})+{d}_{4}{f}_{2}};$$

When there are numbers of incoherent point sources, the hologram intensity distributions described by Eq. (16) are superimposed. From the recorded hologram, the cross-sectional intensity distribution of the object can be obtained by using the Fresnel propagation algorithm.

### Coherent digital holography for cross-sectional quantitative phase imaging

The cross-sectional quantitative phase imaging is accomplished by the off-axis digital holography with a transmission-type configuration, which captures the complex wavefront of the targeted sample without disturbing its physiological condition. The detailed description can be obtained in various papers^4,13–16^. In brief, the off-axis digital holographic microscope is based on the Mach-Zehnder interferometer, in which the coherent laser light source is spatially filtered, collimated, and then divided into the object and reference beams by using a beam splitter. The object beam trans-illuminates the specimen under observation and is collected by the microscope objective, and is magnified by a tube lens. The object beam is allowed to interfere with the reference beam with the use of another beam splitter. The off-axis configuration is created by inducing a small angle between the object wave and the reference plane wave. The phase distribution from the recorded digital hologram is numerically reconstructed after the Fresnel propagation algorithm to the focused plane. Two holograms with and without the specimen are recorded to nullify the aberrations and to obtain the phase distribution of the specimen. This phase distribution corresponding to the specimen is determined by reconstructing both the holograms individually and subtracting their phases.

## Experiments and Discussion

**Figure 3:**
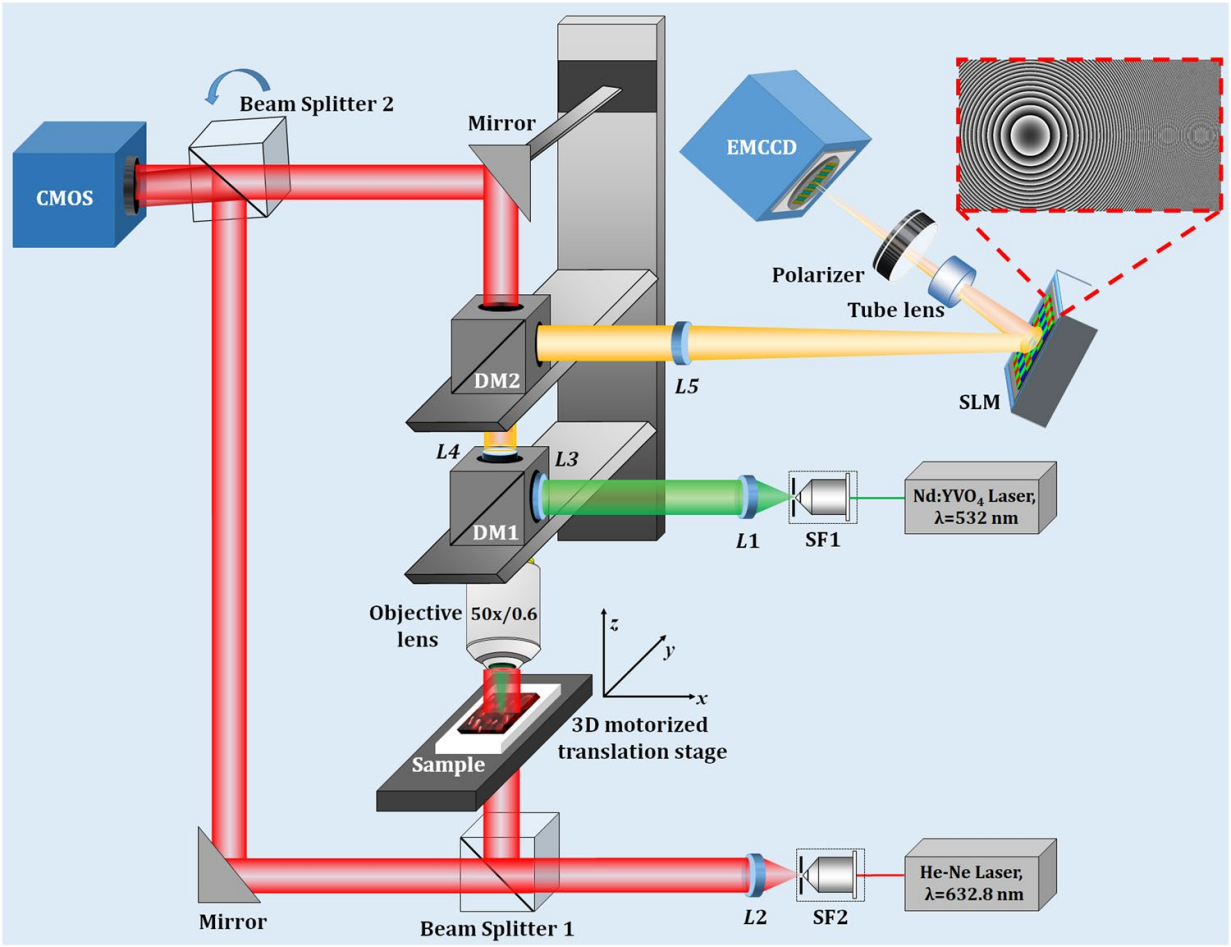
Optical schematic of the proposed multimodal system for the measurement of the cross-sectional fluorescence and the quantitative phase imaging of the specimen.

Figure 3 shows the schematic of the proposed multimodal system with one incoherent digital holographic microscopy and another coherent digital holographic microscopy, working simultaneously for the measurement of the cross-sectional fluorescence imaging and the quantitative phase imaging, respectively. The incoherent digital holographic microscopy system first uses an Nd:YVO_4_ laser (wavelength, λ = 532 nm) as a light source. The laser beam is expanded and filtered using a spatial filter assembly (SF1) and collimated using a collimator (*L*1). This collimated beam, after reflected from a dichroic mirror (DM1, Thorlabs, DMLP567R) entered into the microscope objective (Nikon CF160 TU Plan Epi ELWD, 50×/0.60). The incident light excites the fluorescent beads of size ~10–14 µm (mean size = 10.4 µm, Spherotech Inc.) in an epi-illumination configuration. The fluorescent beads emit the yellow fluorescence with wavelengths ranging from 550 nm to 600 nm. This fluorescent light travels back through the microscope objective, transmits through DM1 and is reflected from another dichroic mirror, DM2 (Thorlabs, DMLP605R). The Fourier transform of the object beam located at the focused plane of the objective lens is projected onto the plane of the phase-mode SLM (Holoeye Pluto, 1920 × 1080 pixels, 8 μm pixel pitch, phase-only modulation) by a 4 *f* relay system (*L*4 → *L*5). Here, the back focal plane of the objective lens is imaged on the phase-mode SLM. A lens function with focal length, *f*_*a*_ = 800 mm and a diffraction grating function with grating period *d*_*h*_ = 300 μm were displayed onto the SLM which generates two distinct wavefronts at respective angles of the incident beam. These two wavefronts are then imaged by a tube lens and allowed to interfere on to the faceplate of the Electron Multiplying charge-coupled device (EMCCD) sensor (Model–iXon 888, sensor format: 1024 × 1024 pixels, pixel size of 13 µm, sensor diagonal of 18.8 mm) and hence form a fluorescent digital hologram. A linear polarizer is placed before the EMCCD sensor to allow the interference of two beams. A band-pass filter centered at 575 nm ± 12.5 nm was placed in front of an EMCCD sensor in order to improve the fringe visibility of the holograms.

The phase imaging is realized by the coherent digital holographic microscopy working in the transmission-type configuration, in which a He-Ne laser (λ = 632.8 nm) is used as an illumination light source. The laser beam is expanded and filtered spatially by a spatial filter (SF2) and collimated by a collimator (*L*2). The collimated laser beam is divided by a beam splitter into two beams: one is the object beam, which trans-illuminates the specimen under study and another is the reference beam. The object beam, after trans-illuminate the specimen, is collected by the microscope objective and collimated by *L4*. The DMs are selected in such a way that the object beam passes through both of them. The object and reference beams are combined at the image sensor plane with the help of another beam splitter, tilted slightly to achieve the off-axis configuration. Note that it is not necessary to put the object beam in the image plane of the image sensor because the digital holographic reconstruction can focus on any plane by changing the propagation distance. The interference pattern is recorded by a CMOS camera (Sony Pregius IMX 249, sensor format: 1920 × 1200 pixels, pixel size of 5.86 μm). In this case, two holograms are recorded: one in the presence of the specimen and another without the specimen. This is because the resultant noise caused by the imperfection of the collimated beams and some aberrations of optical elements can be decreased or eliminated.

**Figure 4:**
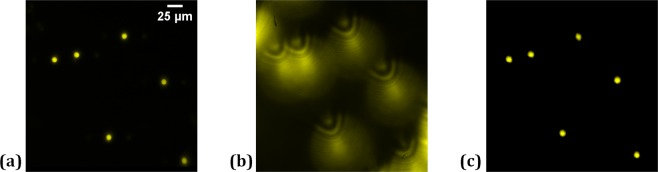
Experimental results of incoherent digital holographic microscopy: (**a**) original focused image of the fluorescent beads, (**b**) corresponding digital hologram obtained by moving the beads by 80 µm along the *z*-direction, and (**c**) reconstructed image of the fluorescent beads.

The performance of the proposed multimodal system was verified by executing various experiments on the fluorescent beads and fluorescent protein-labeled living plant cells. In the first experiment, fluorescent beads, placed on the glass plate mounted on 3D motorized translation stage, are imaged by incoherent digital holographic microscopy, as shown in Fig. 4(a). Afterward, the glass plate is moved in the *z*-direction by 80 µm. A fluorescent digital hologram, as shown in Fig. 4(b), is recorded by projecting a lens function with focal length, *f*_*a*_ = 800 mm and a diffraction grating function with grating period *d*_*h*_ = 300 μm on to the SLM. Pseudocolor according to the central wavelength of the fluorescent light is put on by digital processing. Figure 4(c) shows the reconstructed image of the fluorescent beads retrieved from the recorded digital hologram by the Fresnel propagation algorithm at the reconstruction distance 1020 mm.

**Figure 5:**
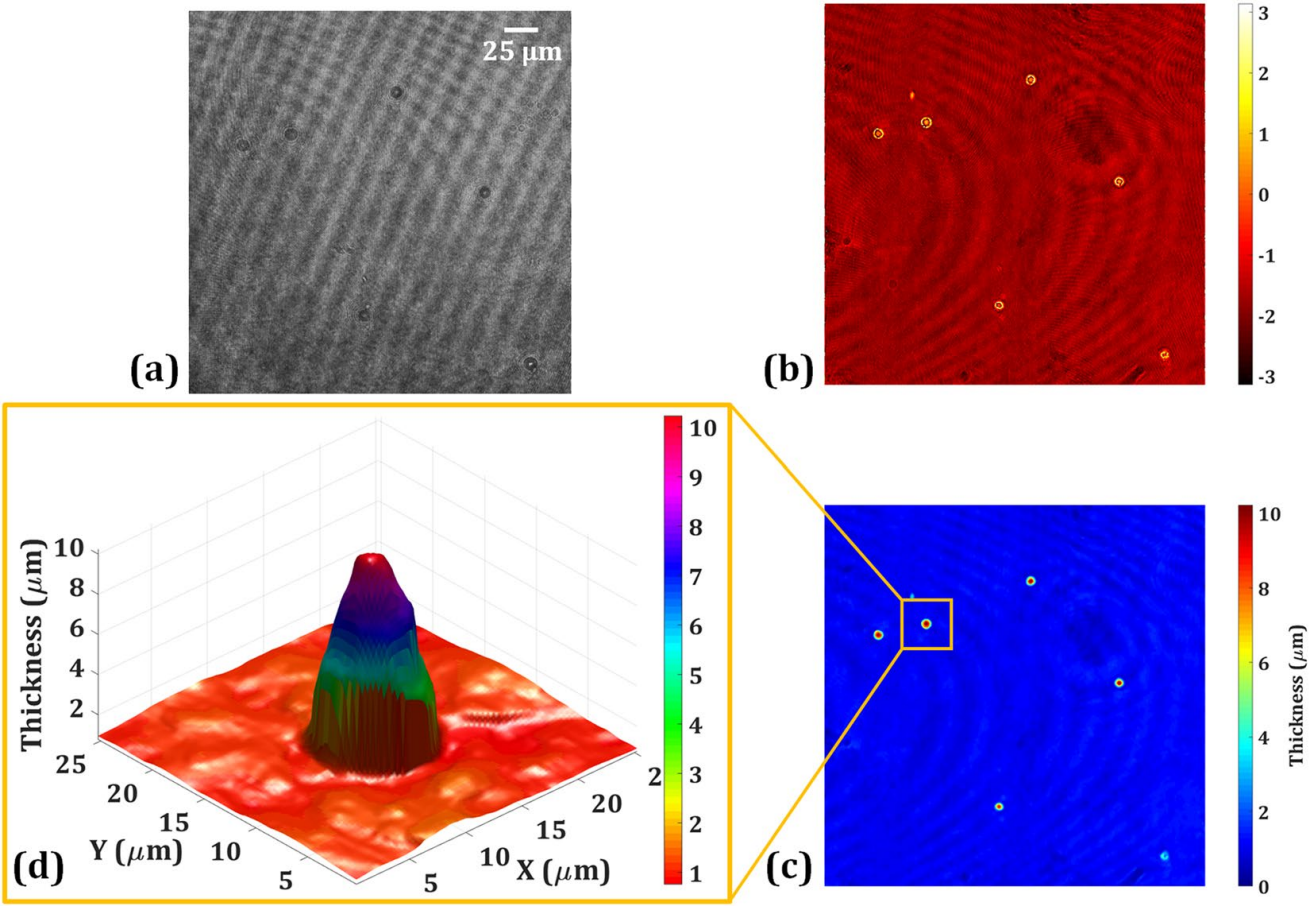
Experimental results of quantitative phase imaging: (**a**) recorded phase hologram in the presence of the fluorescent beads, (**b**) the wrapped phase image, (**c**) thickness map, and (**d**) pseudo-3D thickness profile of the selected bead.

At the same time, the phase microscopic system is also working. Figure 5 shows the phase imaging system’s capabilities by imaging the same fluorescent beads. In this system, two digital holograms are acquired corresponding to the object and without the object. After processing, the quantitative phase image is reconstructed. Figure 5(a) shows the recorded digital hologram in the presence of the fluorescent beads and the reconstructed wrapped phase image of the fluorescent beads is shown in Fig. 5(b). The unwrapped phase map (Δφ) is obtained by using the PUMA phase unwrapping algorithm^41^ and the thickness is measured as, thickness, $h=\frac{\varDelta \varphi \times \lambda }{2\pi \varDelta n}$, where $\Delta n={n}_{s}-{n}_{m}$ with *n*_*s*_ and *n*_*m*_ are the refractive indices of the sample and medium, respectively. Figure 5(c) shows the 2D thickness map of the fluorescent beads and Fig. 5(d) shows the pseudo-3D thickness profile of a selected bead. From Fig. 5(b), we successfully measured the bead size (~10 μm in diameter) along *x*-, *y*-, and *z*-axes. In Fig. 5(b), five beads are clearly seen. This is the phase imaging results of the same fluorescent beads whose fluorescent imaging results are obtained by the fluorescence digital holography and shown in Figs. 4(a,c).

**Figure 6:**
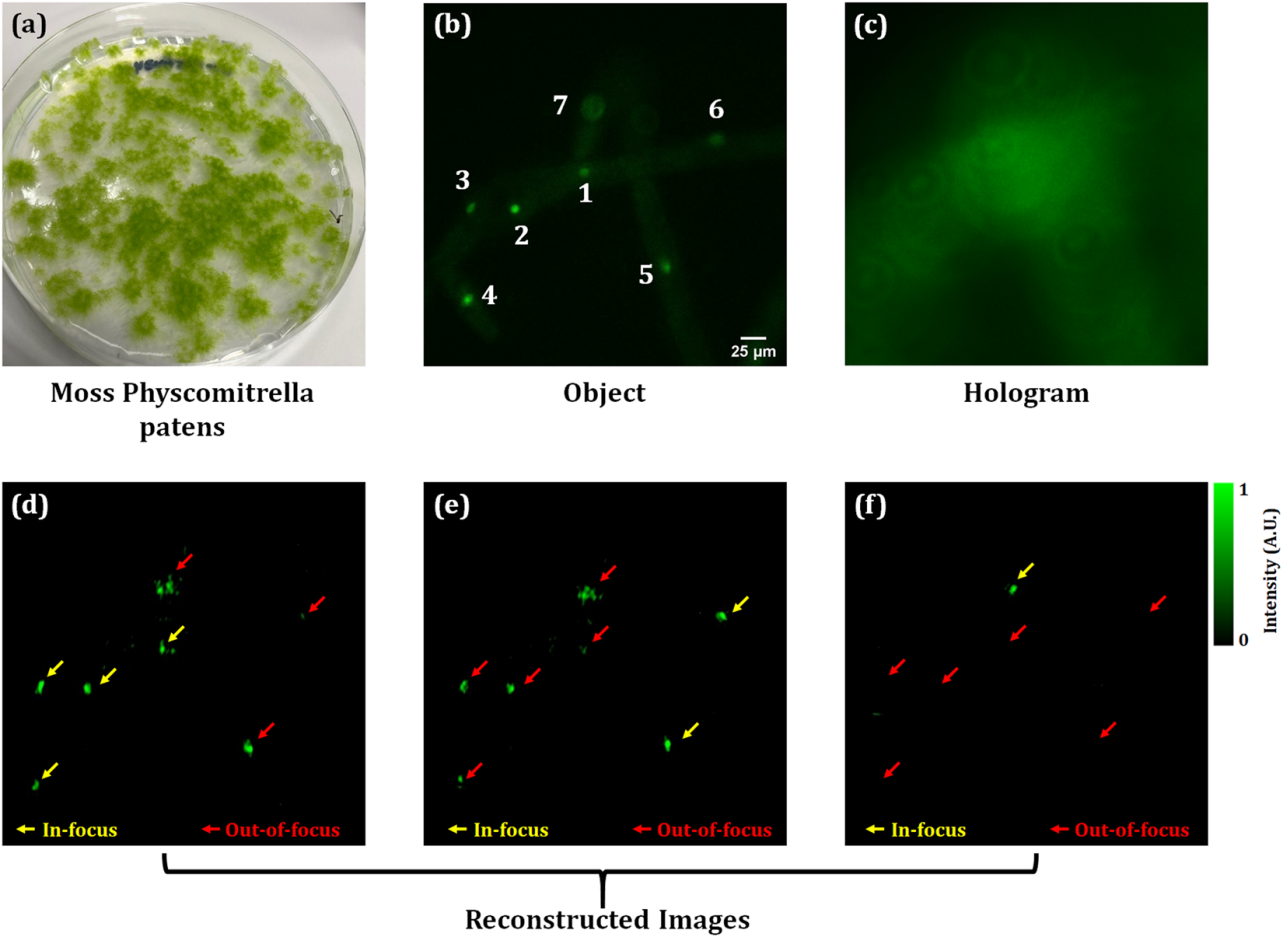
Cross-sectional fluorescence imaging results of living cells of Physcomitrella: (**a**) the photograph of the plant cells, (**b**) the fluorescent image of fluorescent protein-labeled nuclei, (**c**) recorded fluorescent hologram, (**d–f**) the reconstructed images focused on the nuclei in different planes obtained by Fresnel propagation algorithm from the same recorded fluorescent hologram shown in (**c**). The yellow arrows indicate the in-focus nuclei and the red arrows indicate the out-of-focus nuclei.

We experimentally demonstrate the cross-sectional fluorescence and phase imaging capability of the proposed multimodal system on fluorescent protein-labeled living plant cells. In this experiment, we used the protonemata, the hypha-like structure of the moss *Physcomitrella patens* (Physcomitrella), a photograph of which is shown in Fig. 6(a). Physcomitrella is one of the model organisms for molecular, cellular, and developmental biology, due to its sequenced genome, clear cell identity, and compact body size^42^. We thus chose Physcomitrella as the target object of the experiments. We used a transgenic Physcomitrella, where the nuclei are labeled with Citrine Yellow fluorescent protein^40,43^.

A blue laser (λ = 473 nm) is used for excitation of the nuclei labeled with fluorescent proteins. Figure 6(b) shows the fluorescence image of the nuclei of the living plant cells which are distributed in 3D space where nuclei numbered 1–4 are in-focus, nuclei 5 and 6 are out-of-focus by around 10 µm and nucleus 7 is out-of-focus by around 25 µm with respect to the plane of nuclei 1–4, respectively. Figure 6(b) shows the limitation of the conventional full-field fluorescence microscope because it is impossible to obtain all focused images when the plant structure is not flat. By using the proposed common-path off-axis incoherent digital holographic microscope, all focused images to the nuclei can be obtained from one hologram by the numerical wave propagation. Focused position can be determined automatically by evaluating the sharp edge or other criteria. The focused plane corresponding to nuclei 1–4 is moved by 60 µm in the *z*-direction and a hologram is recorded, as shown in Fig. 6(c), by projecting a lens function (with a focal length of 800 mm) and a diffraction grating function (with a grating period of 300 µm) on to the SLM. Figures 6(d–f) show the reconstructed images focused on each nucleus of the plant cells obtained by Fresnel propagation algorithm (in MatLab 2017Rb) at distances 680 mm, 810 mm, and 1050 mm, which are corresponding to 60 µm, 70 µm, and 85 µm, respectively, in the object space.

**Figure 7:**
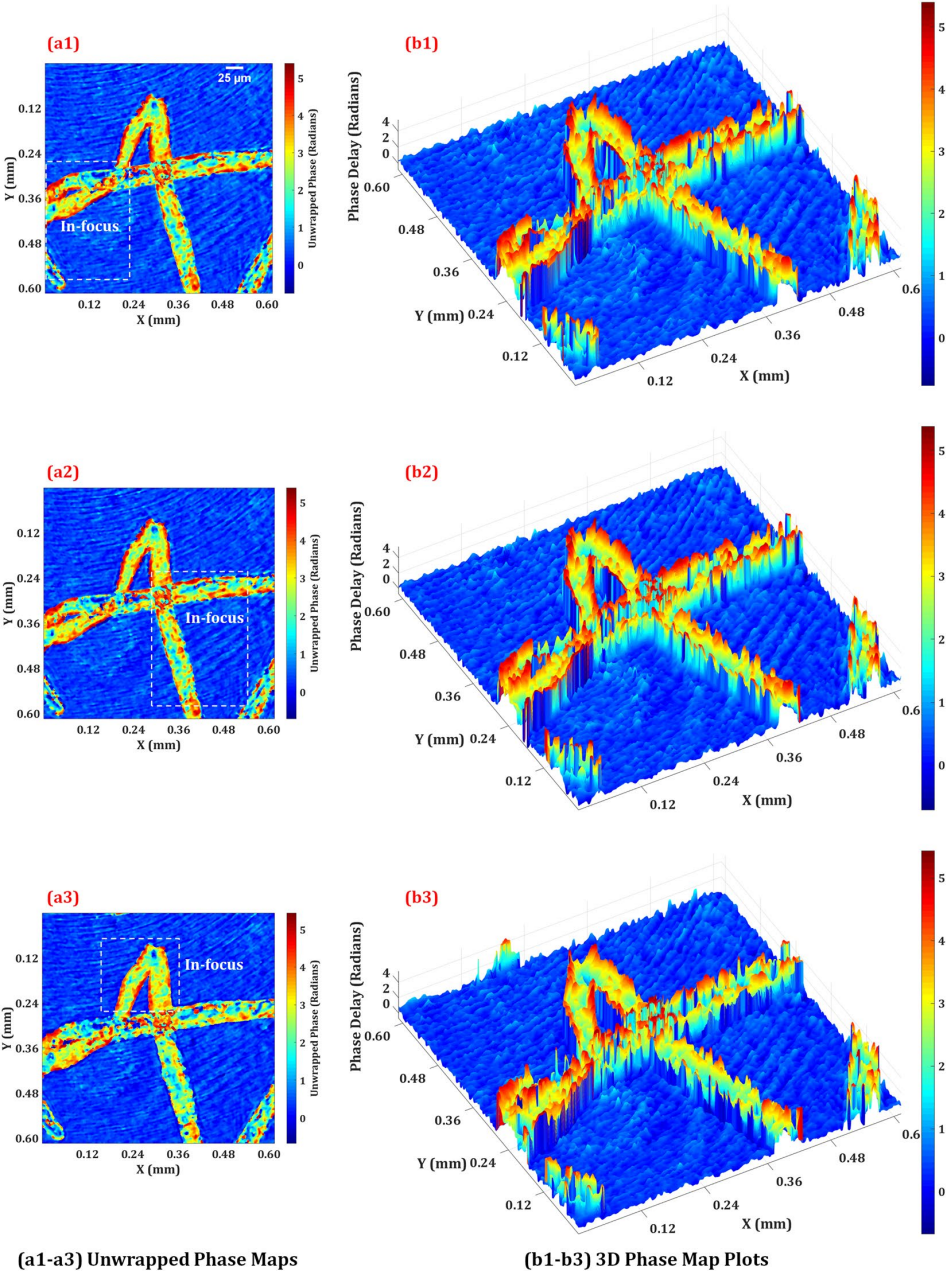
Quantitative phase imaging results of the plant cells: the obtained 2D unwrapped phase maps (**a1–a3**) and corresponding 3D phase plots (**b1–b3**) of the plant cells for the three focused planes corresponding to Figs. 6(d–f) obtained by the Fresnel propagation algorithm from the recorded phase hologram. The dotted rectangular in 2D unwrapped phase maps represent the focused regions.

Figures 7(a,b) show the quantitative phase imaging results of the same plant cells where Figs. 7(a1–a3) show the unwrapped 2D phase maps of the three focused planes of the plant cells and their corresponding 3D phase map plots are shown in Figs. 7(b1–b3). The dotted rectangular in 2D unwrapped phase maps of Figs. 7(a1–a3) represent the focused regions. The reconstruction distance differences between the three planes along the depth directions are 3.5 mm and 11.6 mm which are equivalent to 10 µm and 25 µm, respectively, in the object domain. The obtained unwrapped phase images show the phase shifts are in a range of 0–5 radians. When we assume that the refractive index of the plant cell is 1.36^44,45^, the thickness of the cell is about 17 µm. The obtained thickness is in an appropriate range. There are inhomogeneous phase distributions in the plant cell because of the nucleus and many chloroplasts.

**Figure 8:**
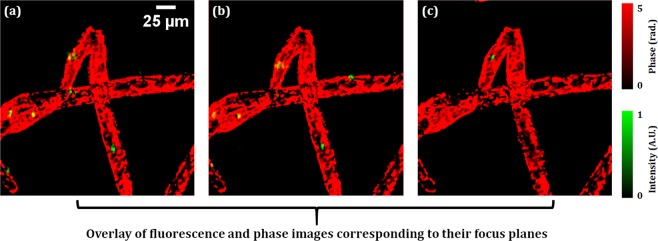
(**a**–**c**) the overlay of the focused fluorescence images of Figs. 6 (**d–f**) and corresponding phase images of Figs. 7(a1–a3).

Figures 8(a–c) show the overlays of the focused fluorescence images and the corresponding unwrapped phase images of the plant cells. From both observations of the fluorescence and phase, the 3D position of the nucleus with the quantitative phase can be retrieved with a single shot. These results show the high potential of our system to observe the 3D behavior of live cells in a single-shot measurement.

**Figure 9:**
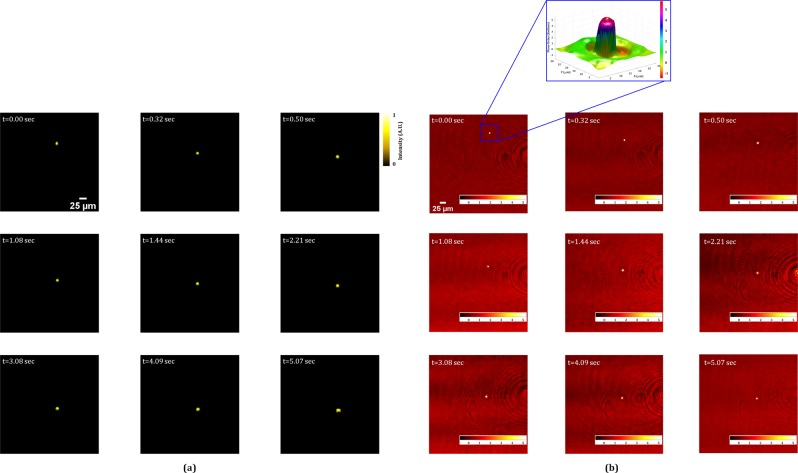
Live fluorescence and phase imaging reconstructions: (**a**) fluorescence images and (**b**) phase images, of a fluorescent bead (inset shows the pseudo-3D phase plot of the bead). Also see Visualization 1 (fluorescence hologram), Visualization 2 (reconstructed fluorescence movie), Visualization 3 (phase hologram), and Visualization 4 (reconstructed unwrapped phase map).

**Figure 10:**
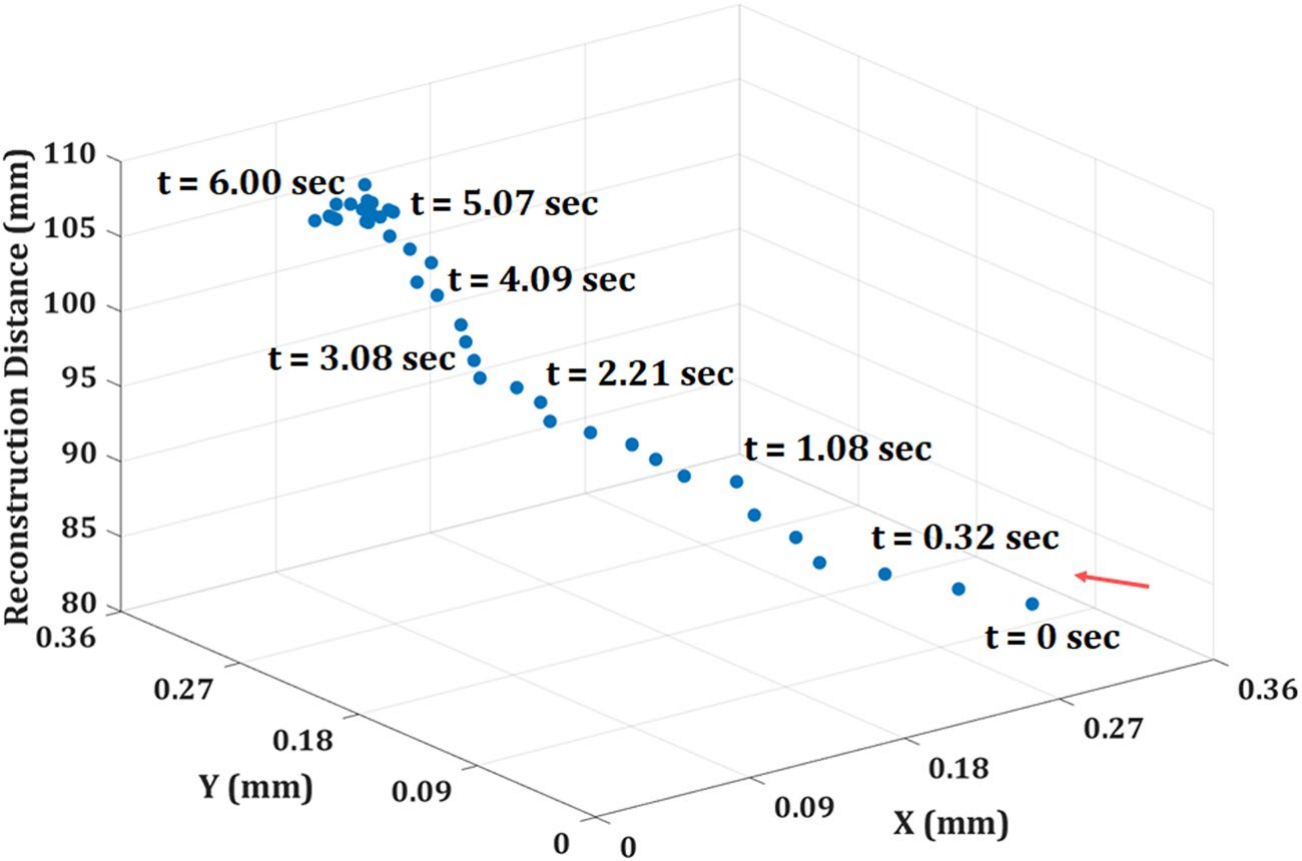
The trajectory of the fluorescence bead.

Furthermore, the multimodal system is applied for the live fluorescence and phase imaging of the moving fluorescent bead in a volume. Figure 9(a) shows the reconstructed fluorescence imaging of moving bead, at progressive time intervals, retrieved from the digital fluorescence holograms recorded by the incoherent digital holographic system. The movie of the holograms of the moving fluorescent bead is shown in Visualization 1. The corresponding movie of the reconstructed fluorescence imaging is shown in Visualization 2. The phase maps of the moving bead, corresponding to Fig. 9(a), are obtained by coherent digital holographic microscopy and are shown in Fig. 9(b) at different time intervals. The movies of the recorded phase holograms and the corresponding reconstructed unwrapped phase maps are shown in Visualizations 3 and 4, respectively. Figure 10 shows the trajectory of the bead. In future work, the time-lapse measurement will be implemented to observe the stem cell formation process in Physcomitrella^46^ via 3D visualization of the simultaneous behaviors of the nucleus and cell structures.

## Conclusion

A new multimodal system is proposed for simultaneous recording and reconstruction of cross-sectional fluorescence and quantitative phase imaging of fluorescent microsphere beads and successfully implemented for the fluorescence and phase imaging of living plant cells labeled with fluorescent proteins. The system comprises of an off-axis Fresnel incoherent digital holographic microscopy and another off-axis coherent digital holographic microscopy working in transmission-mode for recording and retrieving simultaneously the cross-sectional fluorescence images and the quantitative phase of the fluorescent objects. The experimental results presented here reveal the system’s capability for simultaneous investigations of cross-sectional fluorescence and phase information on a single platform. The proposed multimodal system will be the basis for the imaging of rapidly moving vital phenomena in living 3D structures, such as cytoskeleton dynamics^47^, neuronal activation^48^, and morphogenetic flow^49^. Since both the fluorescence and the quantitative phase images of a biological sample can be retrieved from the proposed system, it is possible to extract important biophysical parameters of the sample including its shape and volume, refractive index distribution and the dry mass, etc. Therefore, such multimodality imaging systems could find an immensely beneficial role for improved diagnosis of various diseases. The system could further be upgraded towards a more compact and stable one to provide enhanced image quality and to allow real-time visualization of living biological cells and tissues along with the measurement of various aforesaid intrinsic biophysical parameters.

## Supplementary information

Supplementary Information.

Supplementary Information2.

Supplementary Information3.

Supplementary Information4.

## References

[1] Park Y, Popescu G, Badizadegan K, Dasari RR, Feld MS (2006). Diffraction phase and fluorescence microscopy. Opt. Express.

[2] Pavillon N (2010). Cell morphology and intracellular ionic homeostasis explored with a multimodal approach combining epifluorescence and digital holographic microscopy. J. Biophoton.

[3] Zlotek-Zlotkiewicz E, Monnier S, Cappello G, Berre ML, Piel M (2015). Optical volume and mass measurements show that mammalian cells swell during mitosis. J. Cell Biol.

[4] Quan X, Nitta K, Matoba O, Xia P, Awatsuji Y (2015). Phase and fluorescence imaging by combination of digital holographic microscopy and fluorescence microscopy. Opt. Rev..

[5] Schürmann M (2018). Three‐dimensional correlative single‐cell imaging utilizing fluorescence and refractive index tomography. J. Biophoton.

[6] Chowdhury S, Eldridge WJ, Wax A, Izatt JA (2017). Structured illumination multimodal 3D-resolved quantitative phase and fluorescence sub-diffraction microscopy. Biomed. Opt. Express.

[7] Descloux A (2018). Combined multi-plane phase retrieval and super-resolution optical fluctuation imaging for 4D cell microscopy. Nat. Photon.

[8] Shin S, Kim D, Kim K, Park Y (2018). Super-resolution three-dimensional fluorescence and optical diffraction tomography of live cells using structured illumination generated by a digital micromirror device. Sci. Rep..

[9] Byeon H, Lee J, Doh J, Lee SJ (2016). Hybrid bright-field and hologram imaging of cell dynamics. Sci. Rep..

[10] Zheng J, Zuo C, Gao P, Nienhaus GU (2018). Dual-mode phase and fluorescence imaging with a confocal laser scanning microscope. Opt. Lett..

[11] Yeh LH, Chowdhury S, Repina NA, Waller L (2019). Speckle-structured illumination for 3D phase and fluorescence computational microscopy. Biomed. Opt. Express.

[12] de Kernier I (2019). Large field-of-view phase and fluorescence mesoscope with microscopic resolution. J. Biomed. Opt..

[13] Popescu, G. *Quantitative Phase Imaging of Cells and Tissues* (McGraw-Hill, New York, 2011).

[14] Kim MK (2010). Principles and techniques of digital holographic microscopy. SPIE Reviews.

[15] Yu X, Hong J, Liu C, Kim MK (2014). Review of digital holographic microscopy for three-dimensional profiling and tracking. Opt. Eng..

[16] Park Y, Depeursinge C, Popescu G (2018). Quantitative phase imaging in biomedicine. Nat. Photon.

[17] Popescu G (2008). Optical imaging of cell mass and growth dynamics. Am. J. Physiol. Cell Physiol..

[18] Yang C, Wax A, Dasari RR, Feld MS (2001). Phase-dispersion optical tomography. Opt. Lett..

[19] Charrière F (2006). Cell refractive index tomography by digital holographic microscopy. Opt. Lett..

[20] Fu D (2010). Quantitative dispersion microscopy. Biomed. Opt. Express.

[21] Rinehart M, Zhu Y, Wax A (2012). Quantitative phase spectroscopy. Biomed. Opt. Express.

[22] Rinehart MT, Park HS, Wax A (2015). Influence of defocus on quantitative analysis of microscopic objects and individual cells with digital holography. Biomed. Opt. Express.

[23] Yuste R (2005). Fluorescence microscopy today. Nat. Methods.

[24] Giepmans BN, Adams SR, Ellisman MH, Tsien RY (2006). The fluorescent toolbox for assessing protein location and function. Science.

[25] Lichtman JW, Conchello JA (2005). Fluorescence microscopy. Nat. Methods.

[26] Rahman S. & Lipert, J. Exploration of simple analytical approaches for rapid detection of pathogenic bacteria. Iowa State University (2005).

[27] Pavlova I, Williams M, El-Naggar A, Richards-Kortum R, Gillenwater A (2008). Understanding the biological basis of autofluorescence imaging for oral cancer detection: high-resolution fluorescence microscopy in viable tissue. Clin. Cancer Res..

[28] Kawamoto F (1991). Rapid diagnosis of malaria by fluorescence microscopy with light microscope and interference filter. Lancet.

[29] Buurman EP (1992). Fluorescence lifetime imaging using a confocal laser scanning microscope. Scanning.

[30] Wells, K. S., Sandison, D. R., Strickler, J. & Webb, W. W. Quantitative fluorescence imaging with laser scanning confocal microscopy. In Handbook of Biological Confocal Microscopy, pp. 27–39. Springer, Boston, MA, 1990.

[31] Egeblad M (2008). Visualizing stromal cell dynamics in different tumor microenvironments by spinning disk confocal microscopy. Dis. Model. Mech.

[32] Poon T-C (1985). Scanning holography and two-dimensional image processing by acousto-optic two-pupil synthesis. J. Opt. Soc. Am..

[33] Poon T-C (1995). Three-dimensional microscopy by optical scanning holography. Opt. Eng..

[34] Rosen J, Brooker G (2008). Non-scanning motionless fluorescence three-dimensional holographic microscopy. Nat. Photon.

[35] Kim MK (2012). Adaptive optics by incoherent digital holography. Opt. Lett..

[36] Quan X, Matoba O, Awatsuji Y (2017). Single-shot incoherent digital holography using a dual-focusing lens with diffraction gratings. Opt. Lett..

[37] Rosen J (2019). Recent advances in self-interference incoherent digital holography. Advan. Opt. Photon.

[38] Jang C, Clark DC, Kim J, Lee B, Kim MK (2016). Signal enhanced holographic fluorescence microscopy with guide-star reconstruction. Biomed. Opt. Express.

[39] Quan X (2018). Three-dimensional stimulation and imaging-based functional optical microscopy of biological cells. Opt. Lett..

[40] Kumar M (2020). Common-path multimodal 3d fluorescence and phase imaging system. J. Biomed. Opt..

[41] Bioucas-Dias JM, Valadao G (2007). Phase unwrapping via graph cuts. IEEE Trans. Image Proces.

[42] Kofuji R, Hasebe M (2014). Eight types of stem cells in the life cycle of the moss Physcomitrella patens. Curr. Opin. Plant Biol..

[43] Heikal AA, Hess ST, Baird GS, Tsien RY, Webb WW (2000). Molecular spectroscopy and dynamics of intrinsically fluorescent proteins: coral red (dsRed) and yellow (Citrine). Proc. Natl. Acad. Sci. USA.

[44] Liang XJ, Liu AQ, Lim CS, Ayi TC, Yap PH (2007). Determining refractive index of single living cell using an integrated microchip. Sensors and Actuators A: Physical.

[45] Choi W (2007). Tomographic phase microscopy. Nature Methods.

[46] Li C (2017). A Lin28 homologue reprograms differentiated cells to stem cells in the moss Physcomitrella patens. Nat. Commun..

[47] Murata T (2013). Mechanism of microtubule array expansion in the cytokinetic phragmoplast. Nat. Commun..

[48] Fosque BF (2015). Labeling of active neural circuits in vivo with designed calcium integrators. Science.

[49] Streichan SJ, Lefebvre MF, Noll N, Wieschaus EF, Shraiman BI (2018). Global morphogenetic flow is accurately predicted by the spatial distribution of myosin motors. eLife.

